# SOMEA: self-organizing map based extraction algorithm for DNA motif identification with heterogeneous model

**DOI:** 10.1186/1471-2105-12-S1-S16

**Published:** 2011-02-15

**Authors:** Nung Kion Lee, Dianhui Wang

**Affiliations:** 1 Department of Computer Science and Computer Engineering, La Trobe University, Melbourne, Victoria 3086, Australia

## Abstract

**Background:**

Discrimination of transcription factor binding sites (TFBS) from background sequences plays a key role in computational motif discovery. Current clustering based algorithms employ homogeneous model for problem solving, which assumes that motifs and background signals can be equivalently characterized. This assumption has some limitations because both sequence signals have distinct properties.

**Results:**

This paper aims to develop a Self-Organizing Map (SOM) based clustering algorithm for extracting binding sites in DNA sequences. Our framework is based on a novel intra-node soft competitive procedure to achieve maximum discrimination of motifs from background signals in datasets. The intra-node competition is based on an adaptive weighting technique on two different signal models to better represent these two classes of signals. Using several real and artificial datasets, we compared our proposed method with several motif discovery tools. Compared to SOMBRERO, a state-of-the-art SOM based motif discovery tool, it is found that our algorithm can achieve significant improvements in the average precision rates (i.e., about 27%) on the real datasets without compromising its sensitivity. Our method also performed favourably comparing against other motif discovery tools.

**Conclusions:**

Motif discovery with model based clustering framework should consider the use of heterogeneous model to represent the two classes of signals in DNA sequences. Such heterogeneous model can achieve better signal discrimination compared to the homogeneous model.

## Background

Identification of transcription factor binding sites (TFBS) is fundamental of understanding gene regulations. Binding sites or motif instances are typically 10 ~ 15*bp* in length and degenerated in some positions. They are often buried in a large amount of non-functional background sequences, which causes low signal-to-noise ratio. Hence, using computational approaches to discriminate motif signals from background signals has not always brought satisfactory results. Development of advanced tools is necessary for more accurate motif predictions.

An essence of computational approaches for motif discovery is to search for motifs that are over-represented in the input sequences compared to the background sequences. Motif over-representation can be explained by the existence of segments that have been evolutionarily preserved due to their functional significance to gene regulation. Hence, appearances of motif instances are rather similar to each other despite having variability in some of their positions [1]. Two issues that are closely related to motif discovery problem are: (i) how to construct a model to represent the motifs and, (ii) how to define a suitable search strategy to find putative motifs from the solution space. Position-specific-scoring-matrix (PSSM) [2] and its variations are the most widely used motif model. This model defines the maximum-likelihood estimation on the probability of nucleotide occurrences in every position of a motif. The motif search strategies can be local or global. Local search algorithms begin with an initial guess of a motif model and iteratively refine this model in the search space to maximize a certain criterion. Two examples of such algorithm are MEME [3] (expectation-maximization) and ALIGNACE (gipps-sampling) [4]). The local search approaches find out one motif at a time. Global search algorithms such as clustering based algorithms (e.g., SOMBRERO [5] and MISCLUSTER [6]) and genetic algorithms based algorithms (e.g., GAME [7] and iGAPK [8]) perform simultaneous searches for multiple candidate motifs by exploring the whole solution space.

In this paper, we aim to develop a SOM [9] based Extraction Algorithm (SOMEA) to discover over-represented motifs in DNA datasets. We seek to use SOM to project *k*-mers (i.e. a subsequence with length *k* of DNA sequences) onto a 2-dimensional (2D) lattice of nodes. Through this projection, input patterns (i.e., *k*-mers) with closely related features are projected onto the same or adjacent nodes on the map. Hence, the complex similarity relationships of the high-dimensional input sequence space become apparent on the map. Analysis of selected nodes, therefore, can reveal potential patterns (i.e., motifs) in the dataset.

Previous studies have applied a standard (e.g. [5,10,11]) and hierarchical (e.g. [12]) SOM to discover motifs in protein or DNA sequences. Those studies have made a common assumption that *the motif and the background signals can be analogously modeled by using a homogeneous node model.* This assumption is weak because the two classes of signals have some distinct statistical properties [13]. Hence, homogeneous model of these two signal classes may cause unfaithful map representation and produce clusters with many false positives. The traditional homogeneous modeling of two signal classes implies that, both signals are clusterable under a single type of model. However, mutational events are more rapid in background regions compared to binding regions, causing most of the nucleotide bases in background regions to be random. Thus, they have relatively lower clusterability compared against binding site regions [14]. Therefore, nodes’ vectorial or string [9] based prototypes given by SOM in traditional tools, can represent motifs reasonably well, but do not well suit for background sequences since two different classes of signals are tried to be expressed through a homogeneous modeling. Hence, an alternative modeling approach, preferably a heterogeneous modeling approach, that takes these two signal properties in consideration is necessary.

In the development of SOMEA, we have proposed a hybrid node model to address some of the limitations of current SOM approaches. This hybrid node model is constituted by PSSM [2] and markov chain (MC) [15] model. These two model components perform soft-competition through an adaptive weighting scheme within a node to represent the mixture of signals in it. We hypothesized that, the fitness of each model’s components (i.e., PSSM and MC) with respect to the sequences in a node, is a fuzzy indication of its signal class composition. Heuristic learning rules are proposed in this paper to adjust the model parameters during learning stage. We have evaluated our proposed SOMEA algorithm against several motif discovery tools using real and artificial DNA datasets. Results have shown that, our approach performs significantly better than a state-of-the-art clustering algorithm for motif discovery, named SOMBRERO [5].

## Results and discussion

We now present an experimental evaluation of our SOMEA approach. We have used eight real datasets to compare the performances of our approach against SOMBRERO, MEME, ALIGNACE and WEEDER [16] in terms of sensitivity and specificity. Then, to evaluate SOMEA’s ability in multiple motif discovery, we used five artificial datasets.

For performance quantification, we employed three measures i.e., precision(P), recall(R) and F-measure(F) [17]. They can be computed as: P = TP/(TP + FP), R = TP/Y, F = 2/(1/P + 1/R), where TP, FP, and Y are the numbers of true positives, false positives, and true binding sites in the dataset, respectively. We have considered a predicted site as a true positive if it is overlapped with a true binding site location by at least *x* nucleotides, where *x* is selected according to the length of the true motif consensus.

### Performance on real datasets

The eight test datasets used in this experiments are composed of seven datasets used in [7] and a dataset collected from the Promoter Database of *S. cerevisiae* (SCPD) [18]. Each sequence contains at least one true binding site. These datasets consist of motifs from *Escherichia coli*(CRP), *homo sapiens*(ERE, MEF2, SRF, CREB, E2F, MYOD) and *S. cerevisiae*(GCN4).

SOMEA was run with map sizes that were arbitrarily selected between 10 × 10 to 20 × 20 depending on the size of the dataset. In each case, SOMEA was trained for 100 epochs with a motif length value in [*l* – 3, *l* + 3], where *l* is the known motif consensus length. The top 10 highest ranked motifs according to their MAP score [19] were saved for evaluation purpose. A 3rd order markov chain model [15] was used to compute MAP score. The learning rate parameter was fixed at 0.005 in all the experiments. Whereas, the neighborhood function parameter value, σ was set at 3.0.

For WEEDER, we used the online tool [20] with the following options: sites might appear more than once, both strands, and normal or complete scan. The interesting motifs and their instances that scored at least 90 were used in the evaluation. SOMBRERO was run with the default map sizes and random initialization method. The standalone tool was downloaded from [21]. We evaluated all the “best-motifs” returned by the tools. MEME was run with the “any number” model option and minimum and maximum length value as discussed above. AlignACE was run online [22] with default arguments in most cases.

Table 1 shows recall (R), precision(P) and F-measure (F) rates for a ten run average for each program on the eight real DNA datasets. Comparison shows that, in terms of recall rates, SOMEA performs better than or equally to other tools in four(4) of the eight(8) datasets. Compared to SOMBRERO, SOMEA performs better in terms of recall rates in six(6) of the datasets. Also, SOMEA has higher precision rates in six(6) of the datasets and has better F-measure values in seven of the test datasets(except ERE). Notably, for the MEF2 dataset, SOMEA obtained a much higher precision rate (0.99 vs 0.22) in comparison with SOMBRERO. The performances on all datasets show that, SOMEA achieves significant improvements in the average precision rate (26.9%) and recall rate (13.8%) in comparison with SOMBRERO. This clearly shows that, SOMEA with heterogeneous node model can represent the *k*-mers distribution in DNA sequences better than the algorithms with homogeneous model.

**Table 1:** Evaluation results with comparisons

	SOMEA			SOMBRERO			MEME			ALIGNACE			WEEDER		

	R	P	F	R	P	F	R	P	F	R	P	F	R	P	F
CRP	**0.91**	0.89	**0.9**	0.83	0.43	0.56	0.59	0.88	0.69	0.83	0.98	**0.9**	0.75	0.83	0.79
GCN4	0.69	0.45	0.54	**0.8**	0.41	0.53	0.52	0.52	0.52	0.61	0.62	0.6	0.64	**0.87**	**0.73**
ERE	0.74	0.58	0.65	0.8	0.59	0.67	0.72	**0.82**	0.77	0.75	0.77	0.76	**0.76**	0.54	0.63
MEF2	0.81	**0.99**	**0.89**	0.35	0.22	0.27	**0.92**	0.8	0.85	0.86	0.87	0.86	0.88	0.88	0.88
SRF	0.84	0.74	**0.79**	0.67	**0.83**	0.74	**0.87**	0.72	**0.79**	0.83	0.71	0.77	0.83	0.71	0.76
CREB	**0.89**	0.67	**0.77**	0.83	0.43	0.56	0.59	**0.88**	0.69	0.52	0.66	0.57	0.79	0.71	0.75
E2F	**0.82**	0.64	0.71	0.76	0.67	0.71	0.68	0.64	0.65	0.75	**0.68**	**0.71**	0.89	0.67	**0.76**
MYOD	**0.66**	0.39	**0.49**	0.5	0.32	0.39	0.23	0.38	0.27	0.34	0.31	0.32	0.43	**0.5**	0.46
Average	**0.80**	0.67	**0.72**	0.69	0.49	0.55	0.64	**0.71**	0.65	0.69	0.70	0.69	0.75	**0.71**	0.72

It can be noticed that, SOMEA performance is comparable or better than ALIGNACE, MEME and WEEDER. For example, in terms of F-measure rates, SOMEA produces the best results for five of the eight datasets due to its higher precision rates (note that, both SOMEA and ALIGNACE achieve the same F-measure value for the CRP dataset). SOMEA’s average F-measure value for all datasets (i.e. 0.72) is found better than MEME (0.65), ALIGNACE(0.69) and SOMBRERO(0.55) and equally good as WEEDER(0.72).

It should be noted that, the comparison results between programs cannot be completely fair as every program has its own strengths and weaknesses. For example, some programs might perform rather well for strong motifs; whereas some are designed to discover motifs with certain characteristics (e.g. gapped motifs). The nature of the datasets can be an influential factor to the success of each program. Therefore, the results reported here should serve only as reference.

### Performance on artificial datasets with multiple planted motifs

Practically, we can often find multiple motifs in upstream region of a set of co-regulated genes. These motifs often work as cis-regulatory module to regulate gene expressions. Motif discovery programs should be able to return all of these potential motifs. Local search algorithms, such as MEME, perform a search for single motif at one time; whereas SOMEA and SOMBRERO search for all motifs simultaneously. It is interesting to compare these two strategies.

We have prepared five artificial DNA datasets generated from Annotated regulatory Binding Sites (ABS, v1.0) database [23]. Every DNA dataset has twenty(20) sequences (each with 500bp in length) with three planted real motifs. We run MEME, WEEDER, SOMEA and SOMBRERO five times on each dataset. We asked SOMEA and MEME to return the top 20 motifs for the evaluation purposes. Again, we evaluate all best motifs returned by WEEDER and SOMBRERO.

Table 2 shows the results of comparison between the four algorithms. Overall, SOMEA has the best recall rates in seven(7) of fifteen(15) of the motifs. However, such higher recall rates come at the price of having lower precision rates compared to MEME and WEEDER. Compared to SOMBRERO, SOMEA performs significantly better in most of the datasets in all performance measures. For example, in terms of recall rates, SOMEA is higher in ten(10) of the motifs; whereas, in terms of F-measure values, SOMEA has better results in twelve(12) of the motifs. Hence, it can be observed that, our SOMEA has better signal discrimination ability than SOMBRERO. MEME performs better than SOMEA in terms of average F-measure values in four of five of the datasets. Nonetheless, SOMEA has higher average recall rates in all of the datasets. WEEDER performs poorly in most of the test datasets most likely due to the inability of its scoring function to rank the true motifs highly when planted in the artificial sequences (see WEEDER manual [20]). In summary, both global and local search techniques perform equally well and each strategy has its own strengths and weaknesses. Coupling them could be a feasible approach to enhance motif discovery result.

**Table 2:** Evaluation results with comparisons for multiple motifs datasets

		SOMEA			SOMBRERO			MEME			WEEDER		
R	P	F	R	P	R	R	P	F	R	P	F

Dataset1	CREB	0.43	0.26	**0.33**	**0.44**	0.26	**0.33**	0.20	**1.00**	**0.33**	0.00	0.00	0.00
	MYOD	**0.48**	**0.23**	**0.31**	0.20	0.08	0.11	0.00	0.00	0.00	0.00	0.00	0.00
	TBP	**0.36**	0.21	**0.26**	0.20	0.12	0.15	0.07	**0.50**	0.12	0.00	0.00	0.00
	Avg	**0.42**	0.23	**0.30**	0.28	0.15	0.20	0.09	**0.50**	0.15	0.00	0.00	0.00

	Dataset2	NFAT	0.39	0.27	0.31	0.36	0.21	0.26	**0.44**	**0.78**	**0.56**	0.00	0.00	0.00
		HNF4	0.57	0.40	0.47	**0.63**	0.39	0.48	0.60	0.82	**0.69**	0.40	**1.00**	0.57
SP1		0.50	0.53	**0.50**	**0.53**	0.35	0.42	0.38	**0.54**	0.44	0.00	0.00	0.00
Avg		0.49	0.40	0.43	**0.51**	0.32	0.39	0.47	**0.71**	**0.56**	0.13	0.33	0.19

Dataset3		CAAT	**0.43**	0.21	0.25	0.32	0.17	0.22	0.29	**0.80**	**0.42**	0.00	0.00	0.00
		SRF	**0.70**	0.40	**0.50**	0.59	0.28	0.38	0.29	**0.57**	0.38	0.00	0.00	0.00
	MEF2	0.79	0.45	0.57	0.65	0.31	0.27	**0.80**	0.57	**0.67**	0.27	**1.00**	0.42
	Avg	**0.64**	0.35	0.44	0.52	0.25	0.29	0.46	**0.65**	**0.49**	0.09	0.33	0.14

	Dataset4	USF	0.68	0.39	0.48	**0.73**	0.48	**0.57**	0.41	**0.88**	0.56	0.00	0.00	0.00
		HNF3B	**0.47**	0.25	**0.31**	0.26	0.13	0.17	0.15	**1.00**	0.27	0.00	0.00	0.00
NFKB		0.71	0.47	0.56	0.66	0.46	0.54	**0.80**	0.57	**0.67**	0.33	**1.00**	0.50
Avg		**0.62**	0.37	0.45	0.55	0.36	0.43	0.45	**0.82**	**0.50**	0.11	0.33	0.17

Dataset5		GATA3	**0.61**	0.37	0.46	0.49	0.33	0.36	0.40	0.75	0.52	0.40	**1.00**	**0.57**
		CMYC	0.74	0.47	0.57	**0.89**	0.70	0.84	0.75	**1.00**	**0.86**	0.19	0.75	0.30
	EGR1	**0.66**	0.36	0.47	0.47	0.26	0.33	0.64	**0.81**	**0.72**	0.00	0.00	0.00
	Avg	**0.67**	0.40	0.50	0.62	0.43	0.51	0.60	**0.85**	**0.70**	0.20	0.58	0.29

It should be noted, there are some biases in the comparisons for two reasons. Firstly, both SOMEA and SOMBRERO are rather sensitive to the motif length parameter. As the motif consensuses in a dataset have different lengths, a single run with a fixed length value might not be suited for all motifs. On the contrary, MEME is able to find a length value that suits better each motif. Consequently, in some of SOMEA/SOMBRERO runs, some motifs might appear to be performed better in the experiments. Secondly, the lower precision rates for SOMEA and SOMBRERO could be explained by the fact that the optimal map sizes are not known. Improper map sizes can, to some extend, affect the results for multiple motif datasets.

### Robustness analysis

We have conducted some analysis on the robustness of SOMEA with respect to different map sizes. We have computed recall, precision and F-measure analysis on SOMEA using the eight real datasets for map sizes 10 × 10, 15 × 15, and 20 × 20. Each dataset is run for five times and their average recall, precision, and F-measure is computed.

Table 3 shows the F-measure of eight datasets with different map sizes. It can be seen that, different map sizes affect the performance on the datasets. From the comparisons, it can be noted that the performance of SOMEA and SOMBRERO shows a similar trend. Their F-measure rates reach maximum for most datasets when the map size 15 × 15 is used. The map size 10 × 10 is too small to represent the *k*-mers distribution in the original space for all the datasets. For a smaller map size, naturally the average number of *k*-mers in each cluster increases, hence, the precision rates become lower. In contrast, for a larger map size (i.e., 20 × 20) the precision rates naturally become higher. However, the recall rates can be lower as the true binding site *k*-mers may suffer from sparse distribution among several nodes in the map.

**Table 3:** Comparisons of performance with different map sizes

	SOMEA	SOMBRERO	SOMEA	SOMBRERO	SOMEA	SOMBRERO
	10 × 10		15 × 15		20 × 20	
CREB	0.70	0.41	0.76	0.67	0.72	0.67
CRP	0.81	0.71	0.66	0.71	0.58	0.52
E2F	0.58	0.73	0.69	0.63	0.72	0.67
ERE	0.53	0.42	0.66	0.60	0.61	0.74
GCN	0.41	0.44	0.51	0.52	0.58	0.60
MEF	0.68	0.92	0.91	0.80	0.82	0.44
MYOD	0.32	0.23	0.49	0.42	0.47	0.49
SRF	0.70	0.67	0.77	0.72	0.71	0.71

Showing is the average F-measure from five runs of eight real datasets using three map sizes 10 × 10,15 × 15 and 20 × 20.

The computational time of SOMEA is mainly imposed by three operations: a) finding winner node for each kmer; b) updating winner and its neighboring nodes models; and c) updating node model at the end of an epoch. The time complexity of SOMEA with map size *R* × *C* is *O*((*L* × *R* × *C*) + (*P* × *L*) + (*R* × *C*)), where L is the total length of DNA sequences and *P* is the size of neighborhood during *k*-mers assignment. Here, the *O*(*L* × *R* × *C*) term is due to the computation of finding winning node for every *k*-mer; (*P* × *L*) operations are needed for the computation of updating the temporary model variables during the *k*-mers assignment stage; and (*R* × *C*) operations for updating the node models at the end of an epoch. Self-organizing map based algorithm is known to suffer from heavy computational time due to the global search to simultaneously discover all clusters. We have recorded the execution time of SOMEA for the eight real datasets and found that it has the highest average computational time of 1364s as compared to WEEDER (825s), SOMBRERO (326s), MEME (126.7s) and ALIGNACE (101s). The slower computational time of SOMEA compared to SOMBRERO is due to the fact that we have to update the ∆*M_pssm_* and ∆*M_mc_* parameters (see Methods) for the winner and its neighborhood during *k*-mers assignment (i.e. see Eqs (9) and (10)). In SOMBRERO, the update of node models only occurs at the end of an epoch. Also, some heuristic optimizations are included in it to reduce computational time. It can be observed that, current version of SOMEA requires slightly larger computational time, however, its better sensitivity and specificity performances can offer a good trade-off.

## Conclusions

Motif discovery in DNA datasets is a challenging problem domain because of our lack of understanding of the nature of the data, and the mechanisms to which proteins recognize and interact with its binding sites are still perplexing to biologist. Hence, predicting binding sites by using computational algorithms is still far from satisfaction.

In this paper, we have proposed a SOM based Extraction Algorithm (SOMEA) for simultaneous identification of multiple-motifs in DNA dataset. We have made two main contributions in this work. Firstly, it is shown that, the use of node model that considers the distinct properties of the motif and background signals is helpful in mining DNA motifs. We have proposed a hybrid model that is composed of PSSM and MC model to better represent these two classes of signals. Secondly, it has been highlighted that, clustering based DNA motif mining requires some customizations in the clustering system design, as standard clustering frameworks may not be sufficient. In addition to these, we have proposed heuristic learning rules to update the node model’s parameters during learning.

Many computational motif discovery algorithms have been proposed in the past decade. Like most of these algorithms, SOMEA shares some common challenges that require further investigation. The first is the scalability of the system for large scale dataset such as ChIP sequences. The scalability is the ability of a tool to maintain its prediction performances and efficiency while the size of the datasets increases. To the best of our knowledge, most motif discovery algorithms are not designed to handle large scale datasets. In a recent study [24], using ChIP datasets as benchmark, it is shown that local search techniques such as MEME does not scale well with the increase in dataset sizes. This finding is consistent to an early study by [25]. Currently, SOMEA is not proposed to handle large scale dataset either. However, it can potentially be used to reduce the sequence search space by pre-cluster sequences into lower-dimensional topological space. Then we can perform the motif searches in this lower-dimensional space instead of the original sequence space. It would be interesting to further investigate the feasibility of this search space reduction strategy to enable system scalability.

The second critical issue is the system’s robustness, which relates to the ability of the pattern recognition system to maintain its performance with the changes of parameters and noise in the inputs [26]. Currently, the critical parameters for SOMEA are the map size and the motif length. From our experiences with SOMEA, we found that setting improper map sizes have caused poorer performance. If the map sizes are two small, the precision(recall) rates might be poor(better); whereas if the map sizes are two large, opposite results are expected. Choosing a proper motif length value is important to reveal the true motif patterns. Setting improper length values caused motif discovery algorithms to return only partial motif consensus patterns. We can overcome this shortcoming by running the system with different length values. Through some analysis on the produced results from different runs, we will be able to reveal the true motif consensuses.

In conclusion, clustering biological sequences for motif discovery should consider the use of heterogeneous model to efficiently represent both motif and background signals. We have shown that, our proposed SOMEA using a heterogeneous model, can perform better in terms of sensitivity and specificity than the tools that use homogeneous model.

## System and methods

### Overview

The main idea of our SOMEA algorithm is to use a hybrid node model, where a model is heterogeneously composed of PSSM and MC. We assume each node on the map is a fuzzy composition between a motif signal and background noise. Since we do not have prior knowledge on the type of each node, we use a *soft competitive weighting scheme* for the two components (i.e., PSSM and MC) of each node model. We refer it as intra-node competition. Our framework design is inspired by the fact that, the two sequence classes (i.e. motif and background noise) in the DNA dataset have distinctive properties. Subsequently, it is necessary to represent them using appropriate signal’s models.

SOMEA starts with converting the input DNA sequences (both strands) into a set of *k*-mers using *k* length window shifting through the sequence. Then, the size of the map is defined (user input) and nodes’ model parameters are randomly initialized. Then, the following two learning steps are repeated for each input *k*-mer in the dataset:

1. **Inter-nodes competition:** to find the best matching unit (BMU) of current input *k*-mer *K_j_*.

2. **Models updating:** update model parameters of the BMU including its topological neighborhood.

The two steps above define a recursive regression process [9], where the optimal models parameters are estimated by iteratively applying the *k*-mers to the system. After some training epochs, similar *k*-mers from supposing motif or background class are projected onto the same or adjacent nodes on the 2D grid map. The *k*-mers projected in the vicinity on the map, generally forming clusters. This implies the similarity of their respective features. Once the nodes’ models have been stabilized, we can identify candidate motifs using a motif model evaluation metric.

### Basic concepts and problem formulation

We first give some notations used in this paper, and then describe the SOMEA algorithm. Denoted by *D* = {*S*_1_, *S*_2_,…, *S_N_*}*,* a DNA dataset with *N* sequences. Let a *k*-mer *K_i_* = (*b*_1_*b*_2_…*b_k_*) be a continuous subsequence of length *k* in a sequence, and *i* = 1,…, *Z,* with *Z* is the total number of *k*-mers in that sequence. For a sequence with length *L,* there are *L* – *k* + 1 number of *k*-mers can be produced using *k* length window shifting process.

We can represent a *k*-mer *K* as a 4 × *k* matrix [27]. Let the matrix representation be *e*(*K*) = [*a_ij_*]_4×*k*_, where (*a*_1_*_j_*, *a*_2_*_j_*, *a*_3_*_j_*, *a*_4_*_j_*) = (*A, C, G, T*) and *j* = 1,…, *k*. The matrix has a column *j* representing certain nucleotide *i* at that position *j* in the *k*-mer.

A 2D SOM map is a lattice of *R* × *C* nodes, where *R, C* is the number of rows and columns respectively. Each node *V_ij_*, *i* = 1,…, *R* and *j* = 1,…, *C,* has a subset of *k*-mers assigned to it. For convenience, we use the notation *V_l_* to represent a node, where 1 ≤ *l* ≤ (*R* × *C*). The coordinate of a node *V_l_* in the lattice is expressed as *z_l_* = (*i, j*). Then, each node *V_l_* has a parameterized model Θ*_l_* associated with it.

Let us formulate the clustering based motif discovery task. Clustering on the *k*-mers dataset aims to partition the dataset into a set of non-overlapping clusters {*C*_1_*, C*_2_*,*…*, C_U_*}*,* where each cluster C*_i_* holds a subset of *k*-mers. In our study, each node in the SOM 2D-lattice represents a cluster (i.e. *U = R* × *C*). After forming the clusters, each cluster *C_i_*’s potential is evaluated as true motif using motif model evaluation metric and rank the clusters based on their obtained scores. In SOMEA, we used Maximum A Posteriori score (MAP score) as the model evaluation metric. Then, top *H* highest ranked clusters are selected as putative motifs, and *k*-mers from those clusters indicate the motif locations in the sequences.

### PSSM based motif model *M_pssm_*

We use the Position-Specific-Scoring-Matrix (PSSM) [2] to model the motif signals. The PSSM based motif model, let it denoted by *M_pssm_,* is a matrix, i.e., *M_pssm_* = [*f*(*b_i_, i*)]_4×*k*_, where *b_i_* ∈ {*A, C, G, T*} and *i* = 1,…, *k*. Here, each entry *f*(*b_i_*, *i*) represents the probability of nucleotide *b_i_* in position *i* and . In our SOMEA, the *M_pssm_* for a node *V_l_* can be calculated from the *k*-mers in a node using the maximum likelihood principle, with a pseudo-count value added as under sample correction to the probabilistic model. We follow the Bayesian estimation method for this purpose [28]. The PSSM entries are computed as follows:

*f*(*b_i_, i*) = (*c*(*b_i_, i*) *+ g*(*b_i_*)) */*(*N* + 1), (1)

where *N* is the number of *k*-mers, *c*(*b_i_, i*) is the frequency of nucleotide *b_i_* at position *i* of a set of *k*-mers in a node, *g*(*b_i_*) = [*n*(*b_i_*) + 0.25]/(*N* × *k* + 1) and .

### Markov chain based background model *M_mc_*

In our approach, the background signal is modeled by using the markov chain (MC) model [15]. The MC is a commonly used background signal model to distinguish over-represented motifs from background signals (e.g. in [16,19]). The stochastic and temporal nature of this model can effectively model the complex relationship of the background bases. The MC model assumes that, the probability of occurrence of a nucleotide *b_i_* at position *i* in a DNA sequence is dependent only on the occurrences of *m* previous nucleotides. This relationship can be expressed by the conditional probability *p*(*b_i_*|*b_i_*_–_*_m_*…*b_i_*_–1_), where *b_i_*_–_*_m_*…*b_i_*_–1_ are bases that precede base *b_i_*, and *m* is the markov order. In our approach, the first order MC (i.e. *m* = 1) is used because higher order model usually requires more input data to avoid over-fitting. The maximum likelihood estimation of the conditional probability *p*(*b_i_*|*b_i_*_–_*_m_*…*b_i_*_–1_) is given by [15] as:(2)

where *c*′(*x*) is the number of times sub-sequence *x* found in a set of *k*-mers in a node.

Let us denote *π* (*a, a*′) to represent the conditional probability *p*(*a*′*|a*) of the first order MC, where *a, a*′ ∈ {*A, C, G, T*}. Then the MC transition matrix gives the background model *M_mc_* to be used in SOMEA, i.e., *M_mc_* = [*π*(*a, a*′)]_4×4_ , where .

### Similarity score

A similarity metric is needed for *k*-mers assignment to the nodes during the learning. The score of a *k*-mer *K_j_* = (*b*_1_*b*_2_ …*b_k_*) in respect with the PSSM based model  assigned to node *V_l_*, can be computed as,(3)

Here, *k* is the length of *k*-mer, and *f*(*b_i_, i*) represents the probability of nucleotide *b_i_* in position *i*. Then, the score of a *k*-mer *K_j_* to the MC [15] based model *M_mc_* of node *V_l_* is computed as:(4)

Here, *p*(*b*_1_) is the independent and identically distribution (i.i.d) probability of nucleotide *b*_1_ in current node, which is estimated from the *k*-mers of node *V_l_*.

### Hybrid model

In practice, we are unable to certainly deduce if a SOMEA’s node is a motif or background at any stage of the learning process. Also, before the system converged, the members of a node are likely to be composed of mixed signals. Therefore, neither PSSM or MC based models (*i.e.M_pssm_* and *M_mc_*) alone would satisfactorily model such composition. However, we can weigh the fitness of MC and PSSM models with respect to the *k*-mers in a node. In other words, when a set of *k*-mers fit with a certain model, (i.e., either motif model given by *M_pssm_* or background model given by *M_mc_*), it is more likely that those *k*-mers represent that class. Note that both signal models, can represent signal features from opposite class to some extent.

In this work, we aimed to combine the expression abilities of both of the models (i.e., i.e.*M_pssm_* and *M_mc_*) in an unified mechanism to improve the distinguishing ability of the system, since each node given by SOMEA (or any clustering based approach) contains a fuzzy mixture of motif signals and background signals.

In implementation, we adopted a simple linear weighting scheme to combine these two models for a node *V_l_* as follows:(5)

Equation (5) gives a linear combination of the two models to produce a heterogeneous model for a node *V_l_*. Here, λ is a scaling factor, for simplicity default value of λ is set as 0.5. If a *k*-mer *K_j_* gets a higher score by this heterogeneous model based scoring Θ*_l_*(*K_j_*), that indicates *K_j_* has a better fit to the combined model of node *V_l_*.

### Motif ranking

Once the SOMEA is stabilized after training, we have to perform an evaluation on the nodes in order to identify the most prominent candidate motifs. The candidate motifs can be identified using either motif evaluation metric or statistical significance value. These metrics usually require the use of background sequences model for computation.

In this work, we adopt the Maximum A Posteriori score (MAP score) [19] for motif ranking. The MAP score measures the conservation property of a motif with respect to the species background sequences [19]. Since, rare motifs in the background can achieve a higher MAP score, this measure can be used to distinguish a true motif from false ones based on their scores ranking. The background sequences can be modeled by using the markov chain model generated from the intergenic sequences of a species under study. This model can be used to assign a probability of a *K*, namely , under the background model given by . The MAP score of a node *V_l_* can be calculated as follows:(6)

where *N_l_* is the number of *k*-mers in node *V_l_* and  refer to background probability of a *k*-mer *K* in respect with background model  .can be written as,(7)

Here, *m* is the Markov chain order, *k* is the length of *k*-mers, *p*(*b*_1_, *b*_2_,…, *b*_m_) is the probability of subsequence *b*_1_,*b*_2_*,* …, *b_m_,* and *p*(*b_i_|b_i_*_–_*_m_, b_i_*_–_*_m_*_+1_,…, *b_i_*_–1_) is the conditional probability of the subsequence *b_i_* under *b_i–m_, b_i–m_*_+1,…,_*b_i_*_–1_ occurrence constraints. For instance, using the 3rd order model, the probability of the sequence *ATGCG* can be calculated as: . This background probability is usually pre-computed on the sequences of interest. In Eq (6), *E*(*V_l_*) is the Shannon’s entropy, that can be written as,(8)

Here, *f*(*b*_i_, *i*) is the probability of nucleotide base *b_i_* ∈ {*A, C, G, T*} to occur in *i*-th position of the PSSM.

## Algorithm

In this Section, we describe our SOMEA learning algorithm, which includes the similarity metric used for *k*-mer assignments, model parameters adaptation, and the finding of BMU for a *k*-mer. According to [29], any arbitrary set of items, for which a similarity or distance measure between its elements is definable, can be mapped onto the SOM grid in an orderly fashion. Hence, the standard SOM learning algorithm is applicable for our purposes with some modifications.

### Adaptation process

We opted for the more speed efficient batch training scheme to update the nodes’ model parameters. This method delays the update of the model parameters at the end of an epoch. Heuristic rules are proposed to update each node’s PSSM and MC model parameters. We associate each node with three computing components including: two matrices ∆*M_pssm_*, ∆*M_mc_* and a counter *r*. Let  be BMU of an input *k*-mer *K* = (*b*_1_,*b*_2_,…,*b_k_*). Denoted ∆*M_pssm_* = [∆*f*(*b_i_, i*)]_4×_*_k_* for *b_i_* ∈ {*A, C, G, T*} and *i* = 1,… ,*k*. Similarly, let ∆*M_mc_* = [∆*π*(*a, a*′)]_4×4_ for *a, a*′ ∈ {*A, C, G, T*}. We initialize all entries in both matrices ∆*M_pssm_* and ∆*M_mc_* as 0. Also let *r* = 0. Once a winning node for a *k*-mer *K* is found, the matrices of a node  are updated as follows.(9)(10)

where  is an entry of the binary matrix *e*(*K*), *count*(*a, a*′) is the frequency of di-nucleotide (*aa*’) in *k*-mer *K* and *h* is a neighborhood function. The neighborhood function *h* is defined as(11)

where σ is the variance whose value is fixed throughout the learning stage. We also update *r = r* + 1. Upon completion of an epoch, all nodes’ model parameters will be updated as follows:(12)(13)

where *η* is the learning rate and *f*(*b_i_, i*) and *π* (*a, a*′) is defined in Eq (1) and Eq (2) respectively. Note that, in the computation of Eq (12) and Eq (13), we first compute *f*(*b_i_, i*) and *π* (*a, a*′) using the current set of kmers assigned to a node.

It is also necessary to update the weighting parameters *α* and *β*. Assuming a set of *N_l_k*-mers {*K*_1_ …, *K_Nl_*} is assigned to a node *V_l_* at the end of an epoch, the weighting parameters update equations are(14)

and

*β_new_* = 1* – α_new_*. (15)

### Training

Assuming a set of *k*-mers *X* is available. The high-level training algorithm for SOMEA is as follows.

1. **Inputs.***k*-mer length *k*, number of top motifs to return in the results *H,* markov chain background model, and DNA sequences.

2. **Architecture setup.** The SOMEA lattice size (*U = R* × *C*)*is* arbitrarily chosen. The default size is 10 × 10. Each node’s model, Θ*_i_*, is initialized with random values.

3. **Training.**

Let the BMU index for a *k*-mer K is *q*(*K*).

**for** epoch=1 to max_epoch **do**

**for** each *K* ∈ *X***do**

•Compute Θ*_i_* (*K*),∀*i* = 1, …,*U*.

•Find the BMU of *K* as 

•Assign *k*-mer *K* to node *q*(*K*).

•Update ∆*M_pssm_*, ∆*M_mc_*, *r* of node *q*(*K*) and its neighboring nodes.

end for

Update model parameters of all nodes using Eqs (12) and (13).

end for

4. **Finalizing.**

(a) Compute the MAP score *F*(*V_i_*),∀*i* = 1,…,*U*.

(b) Rank *V_i_* according to their MAP score values.

(c) Save the top *H* ranked *V_i_* as result.

## Author details

Intelligent Search and Discovery Laboratory, Department of Computer Science and Computer Engineering, La Trobe University, Melbourne, Victoria 3086, Australia.

## Authors' contributions

DH conceived the idea of this study and gave the direction on the framework and experimental studies. Under DH supervision, NK implemented the algorithm and carried out the experimental studies. NK drafted the manuscript and amended by DH. Both authors agreed on the final manuscript.

## Competing interests

The authors declare they have no competing interests.
